# DNA Barcoding in Pencilfishes (Lebiasinidae: *Nannostomus*) Reveals Cryptic Diversity across the Brazilian Amazon

**DOI:** 10.1371/journal.pone.0112217

**Published:** 2015-02-06

**Authors:** Denise Corrêa Benzaquem, Claudio Oliveira, Jaqueline da Silva Batista, Jansen Zuanon, Jorge Ivan Rebelo Porto

**Affiliations:** 1 Laboratório de Genética Animal, Instituto Nacional de Pesquisas da Amazônia, Av. André Araújo, 2.936, Petrópolis, CEP 69067-375, Manaus, AM, Brazil; 2 Instituto de Biociências, Departamento de Morfologia, UNESP, CEP 18618-970 Botucatu, SP, Brazil; 3 Laboratório de Fisiologia Comportamental e Evolução, Laboratório Temático de Biologia Molecular, Instituto Nacional de Pesquisas da Amazônia, Av. André Araújo, 2.936, Petrópolis, CEP 69067-375, Manaus, AM, Brazil; 4 Laboratório de Sistemática e Ecologia de Peixes, Instituto Nacional de Pesquisas da Amazônia, Av. André Araújo, 2.936, Petrópolis, CEP 69067-375, Manaus, AM, Brazil; Chinese Academy of Fishery Sciences, CHINA

## Abstract

*Nannostomus* is comprised of 20 species. Popularly known as pencilfishes the vast majority of these species lives in the flooded forests of the Amazon basin and are popular in the ornamental trade. Among the lebiasinids, it is the only genus to have undergone more than one taxonomic revision. Even so, it still possesses poorly defined species. Here, we report the results of an application of DNA barcoding to the identification of pencilfishes and highlight the deeply divergent clades within four nominal species. We surveyed the sequence variation in the mtDNA cytochrome c oxidase subunit I gene among 110 individuals representing 14 nominal species that were collected from several rivers along the Amazon basin. The mean Kimura-2-parameter distances within species and genus were 2% and 19,0%, respectively. The deep lineage divergences detected in *N. digrammus, N. trifasciatus, N. unifasciatus* and *N. eques* suggest the existence of hidden diversity in *Nannostomus* species. For *N. digrammus* and *N. trifasciatus*, in particular, the estimated divergences in some lineages were so high that doubt about their conspecific status is raised.

## Introduction

Neotropical ichthyofauna is extremely large and diverse based on the latest survey of the diversity of freshwater fishes of Central and South America [1], it includes 71 families encompassing 4,475 known valid species. Moreover, the authors estimate there are 6,000 species in the neotropics, close to half the number of freshwater fish worldwide.

The Amazon, home of more than two thousand freshwater fish species, is well known as a diversity hotspot. The complex evolutionary history of Amazonian organisms, including fishes, is gradually beginning to be better understood. Postulated reasons for the origin and evolution of Neotropical fish richness have included tectonic movements and Andes orogenesis leading to the rise and fall of biogeographic barriers, rates of speciation, extinction, and taxa dispersal, and the great antiquity of the Amazonian ecosystem as a whole that dates to the early Cenozoic and Cretaceous periods [2–3].

The identification of Amazonian fish species is a challenging using only morphology as a tool, because fish taxonomists face difficulties daily in the process of identifying species. In this context, there are several debates and statements that molecular data should be encouraged as a supplement to species description and diagnosis, but should not replace morphological data [4–9]. Indeed, species discriminations or descriptions involving the cytochrome c oxidase subunit I (*COI*) appear to be a trend in fish taxonomy [10–16].

The Barcode of Life, referred to as DNA barcoding, has recently emerged as a way to identify species by minimal DNA sequences. While this approach offers all of the advantages of conventional PCR and DNA sequencing, it also connects bioinformatics with the taxonomic identifications in museum and biological repositories, implement a modern and integrated system to ensure that newly reported cryptic species will be described following their discovery [17–18]. This global identification system, based primarily on nucleotide sequences, has been applied to several organisms, and many published works have demonstrated the efficacy of this methodology for species identification [19–27].

By using DNA barcoding, the identification of fish at the species level ranges from 98–100% [18, 28–29] and the discriminatory power of identification has been 93% in freshwater fish and 98% in marine fish species [30].

This worldwide effort often reveals that species richness is still underestimated. As an indicator of cryptic speciation, at least two delimitations have been employed: the “10X rule” and the “cutoff value.” In the first, barcoded specimens from the same nominal species diverge by 10 times or more from the mean intraspecific variability of the group. The “barcode gap”, that is, the divergence among organisms belonging to the same species being much smaller than the divergence among different organisms, has been clearly demonstrated in different several. In the second, individuals with a genetic distance of 2% or more are considered much more likely to be congeneric than conspecific [30].

The fishes of the family Lebiasinidae constitute a Neotropical group with approximately 76 valid species, and they are widely distributed throughout South and Central America [31]. Phylogenetic relationships among the seven genera of the group were examined in 32 representatives of Lebiasinidae (A. L. Netto-Ferreira, unpublished- data). Belonging to this family, *Nannostomus* is comprised of colorful ornamental species distributed throughout different countries in the Amazon Basin such as Colombia, Venezuela, and the Guyanas in the north; Brazil and Bolivia in the south; Peru in the west; and Brazil in the east [32–34]. Most species are slender and, pencil-shaped, ranging in size from 1.5 cm to approximately 7 cm in length, and highly are prized in the aquarium industry [35]. The species typically inhabit the shorelines of small creeks, streams, pools, lakes, and flooded forests, rarely being found in the open primary channels of Amazonian rivers. In these locations, they are typically found in shallow water, living among aquatic plants, leaf litter, and floating meadows [36–37].

Despite its economical importance, little is known about biology or history of life of *Nannostomus* species. However, In Amazonian streams, they graze on algae on submerged trees and dead trunks, and feed on small invertebrates. It also provides food for animals at higher trophic levels developing an important role in the maintenance of the functioning and health of the whole aquatic system [38].

Popularly known as the pencilfish, *Nannostomus* has 20 taxonomically valid species [31]. It is only genus to have undergone two taxonomic revisions, but other poorly defined species complexes probably remain in need of revision, including *N*. *beckfordi* Günther, *N*. *eques* Steindachner, *N*. *marginatus* Eigenmann, and *N*. *trifasciatus* Steindachner (A. L. Netto-Ferreira, unpublished data). Many of the species have conspicuous color variations depending on their geographic location. Indeed, some of these color variants have been described as separate species in the past [39–42].

Recently, molecular analyses were conducted in two *Nannostomus* species (*N*. *eques* and *N*. *unifasciatus*), both of which are widely distributed throughout the Amazon basin [43–44]. Strikingly, the divergence time analyses of the lineages of both species allowed the authors to estimate an approximate divergence time in the middle Pliocene for lineages in *N*. *eques* (around 2.9 Mya) and in the late Miocene for *N*. *unifasciatus* (around 8.4 Mya). The presence of distinct phylogroups in both species have been observed in representatives from the Negro River Basin, showing that due to hidden diversity, those species should not be treated as having a single stock for management purposes. Such data may be useful in regulating the exploitation of fish species by the aquarium trade [43–44].

Given the biological and economic importance of these species, and as part of a study of the molecular phylogeny of *Nannostomus*, we report herein an application of DNA barcoding to the identification of 12 species of pencilfishes and highlight the deeply divergent clades detected within several nominal species, including the presence of additional cryptic species.

## Materials and Methods

### Ethics statement

This survey was carried out in strict accordance with the recommendations by the National Council for Control of Animal Experimentation and the Federal Board of Veterinary Medicine. The protocol was approved by the Committee on the Ethics of Animal Use of the Instituto Nacional de Pesquisas da Amazônia (041/2012).

All specimens for this study were collected in accordance with Brazilian laws under a permanent scientific collection license issued in the name of Dr. Jorge I.R. Porto and approved by the Brazilian Institute of Environment and Renewable Natural Resources (IBAMA) through the System Authorization and Information on Biodiversity (SISBIO #11489-1).

### Sampling data

We sampled 14 species of *Nannostomus* (*N*. *beckfordi*, *N*. *britiskii*, *N*. *digrammus*, *N*. *eques*, *N*. *espei*, *N*. *harrisoni*, *N*. *limatus*, *N*. *margnatus*, *N*. *marylinae*, *N*. *morthenhaleri*, *N*. *nitidus*, *N*. *rubrocaudatus*, *N*. *trifasciatus and N*. *unifasciatus*). After, each specimen was identified, it is anesthetized by immersion in Eugenol in water and preserved in 96% ethanol for the molecular studies. Upon identification, morphological vouchers were deposited in the Zoological Collection at the National Institute for Amazonian Research, INPA, Manaus, Brazil.

**Fig 1:**
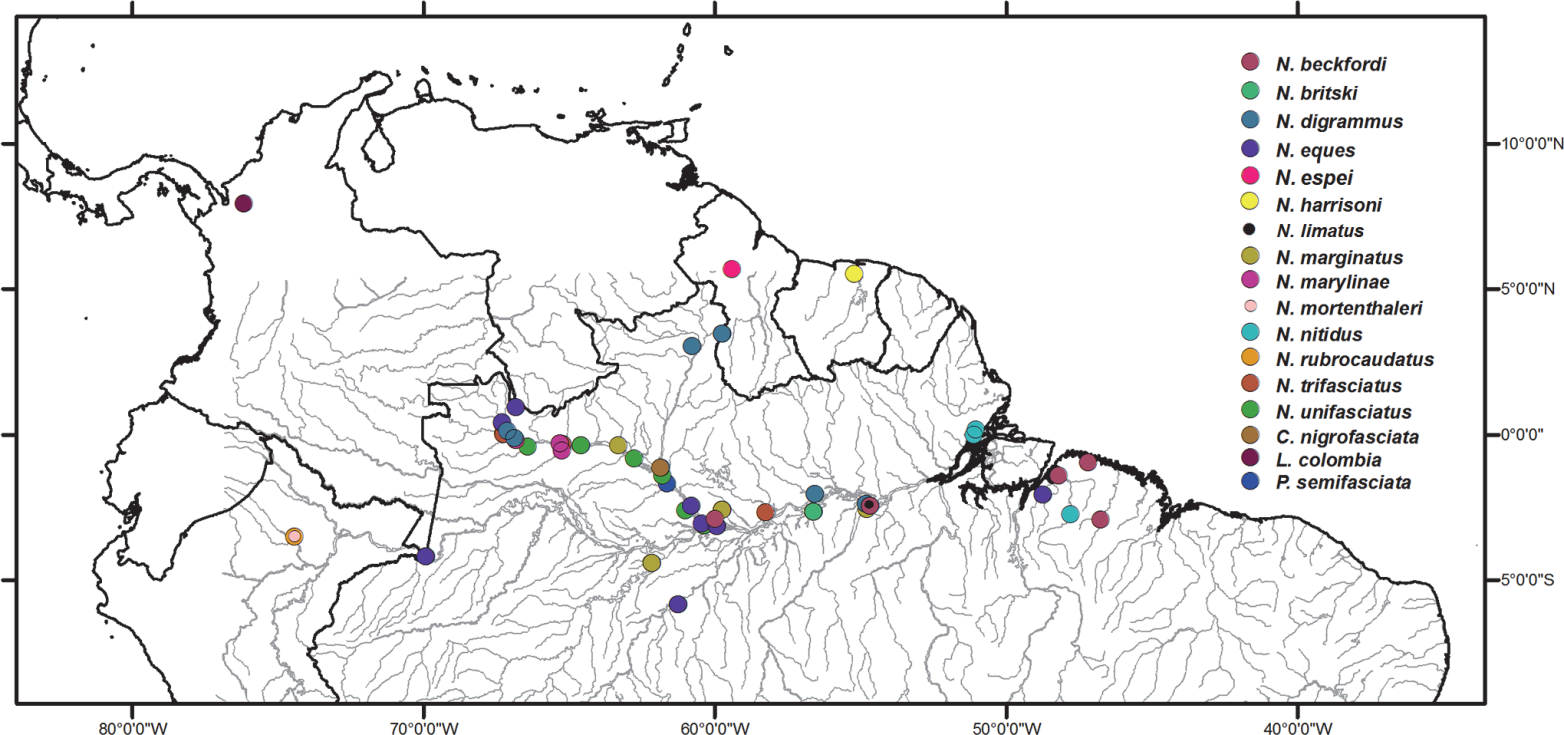
Map of the study area showing the sampling sites the pencilfishes collected in this study.

Sampling sites were chosen at distant points in the Amazon basin in order to cover at a minimum, the distribution range of the species [33] (Fig. 1). Two lebiasinids were used as outgroups (*Lebiasina colombia* and *Copella nigrofasciata*) because they are members of each of the two subfamilies in Lebiasinidae.

All specimens were identified with the help of taxonomists and identification keys [34,37] end all procedures complied with the recommendations of local ethics committees. Voucher specimens were deposited in the collection of the Instituto Nacional de Pesquisas da Amazônia, Manaus, AM, Brazil.

### Extraction, amplification, and DNA sequencing

Genomic DNA was isolated from muscle tissue based on standard techniques and protocols [45]. The partial mitochondrial *COI* gene (648pb) was amplified by PCR using a combination of primers FishF1, FishR1, FishF2 FishR2) [20] or cock-tail C_VF1LF_t1- C_VR1LR_t1 under conditions previously described [46].

Polymerase chain reactions were performed in a total volume of 15μL (∼10–50 ng DNA template, 1X buffer (750 mM Tris-HCl, pH 8.8, 200 mM (NH_4_)_2_SO_4_), 1U Taq polymerase (Thermo Scientific, Waltham, USA), 0.2 mM dNTPs, 0.2 μM of each primer, 2 mM MgCl_2_, and ultrapure water. PCR cycling was performed with the initial denaturation for 2 min at 95°C followed by 35 cycles of 30s at 95ºC, 30s at 52ºC-54ºC, 1 min at 72ºC and with a final extension for 10 min at 72ºC. PCR products were resolved on 1% agarose gels and purified using polyethylene glycol 8000 (USB, Cleveland, USA). The bi-directional sequencing was performed utilizing an ABI BigDye TM Terminator v.3.1 Cycle Sequencing Ready Reaction Kit and an ABI 3130xl DNA Analyzer (Applied Biosystems, Foster City, USA).

Data sequences, collection sites, primers details and trace files were submitted to the Barcode of Life database (BOLD; http//www.boldsystems.org) in under project “Barcoding of Lebiasinids.”

### Data analysis

Consensus sequences for the COI gene were generated using the BioEdit program [47], and after editing the sequences, the final matrix was 574bp.

All sequences were analyzed using MEGA 5 to check the occurrence of deletions, insertions, and stop codons. Search tools with local alignment were used to identify the sequence in GenBank and the BOLD. Sequences were aligned using Clustal W [48], and the program DnaSP version 4.0 [49] was used to determine the nucleotide composition, number of polymorphic sites, and haplotypes diversity.

The genetic distance among and within observed clusters was calculated using the Kimura-2-parameter (K2P) model. A Bayesian phylogenetic analysis was conducted using MrBayes 3.2 [50]. For this analysis, Markov chain Monte-Carlo sampling was conducted every 120,000 generations until the standard deviation of split frequencies was <0.01. A burn-in period equivalent to 25% of the total generations was necessary to recapitulate the parameter values and trees. The parameter values were evaluated based on 95% credibility levels to ensure a sufficient number of generations had been run for the analysis. A neighbor-joining (NJ) tree of K2P distances was created to provide a graphic representation of the relationships among specimens and clusters with MEGA 6.0 [51]. Bootstrap resampling [52] was applied to assess the support for individual nodes using 1000 pseudo-replicates. The program Haploviewer[53] was used to construct a tree-based haplotype network. Independent networks were regarded as unconfirmed candidate species.

## Results

*COI* sequence data were obtained for 110 specimens of *Nannostomus* representing 14 species-level taxa. A mean of 8 individuals (range 1–17) represented each species, with only *N*. *harrissoni* represented by a single specimen.

The amplified product was approximately 650 bp but only 573 nucleotides were considered for this analysis, of which 234 were variable and parsimony informative. These variations defined 68 haplotypes ranging from 1–5 individuals per haplotype. At no time was a detected haplotype shared between different species. There were no deletions, insertions, or stop-codons. As expected for fish *COI*, the nucleotide composition showed a CT bias (means C = 24.0%, T = 32.7%, A = 25.9%, G = 17.3%) within *Nannostomus*.

**Fig 2:**
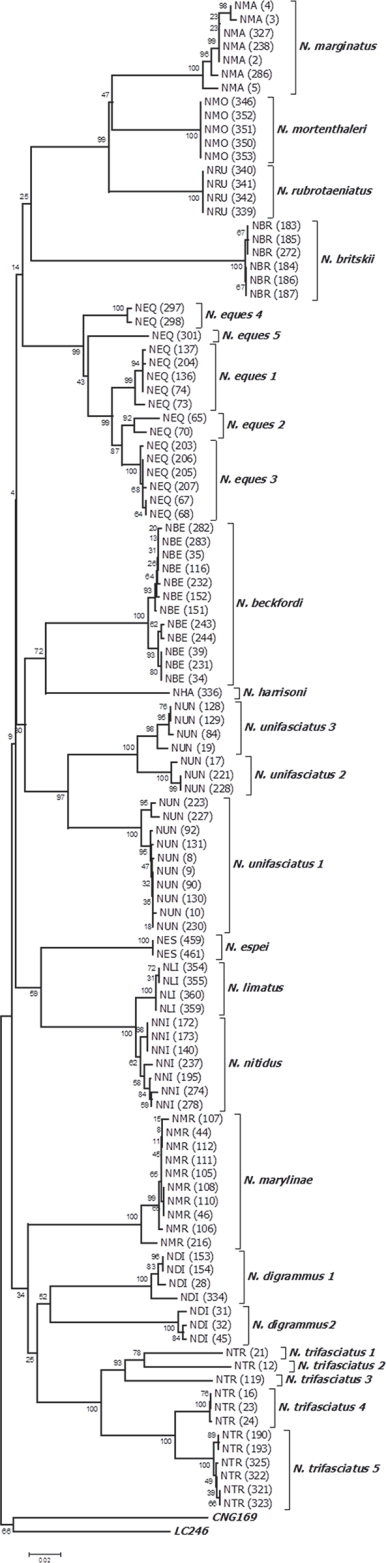
Neighbor-joining tree of 110 mitochondrial cytochrome oxidase subunit I gene sequences from 14 *Nannostomus* species and two outgroups using Kimura 2-parameter. Deep conspecific divergences shown by 4 species (*N*. *digrammus*, *N*. *trifasciatus*, *N*. *unifasciatus* and *N*. *eques*) are identified as distinct lineages. Bootstrap values based on 1000 replicates are indicated at the branches.

The results indicated that species could be discriminated by the DNA barcode approach since the samples of distinct species were represented by a unique haplotype, a single tight cluster of haplotypes, or distinct clusters of haplotypes in neighbor-joining tree (Fig. 2).

The mean genetic distances (Kimura—2-parameter distances) among different *Nannostomus* species ranged from 2.2% to 22.5% (Table 1). The congeneric distance values found in *Nannostomus* were high, with an overall mean of 19. 0% ± 1. 3%, with values >22% between *N*. *britski* and its congeners and 2.2% between *N*. *limatus* and *N*. *nitidus* (Table 1).

**Table 1:** Kimura 2-parameter genetic divergence values in 14 *Nannostomus* species.

	K2P divergence genetic (%)			
	Minimum	Mean	Maximum	SE
Conspecific	0	2,8	9.80	2,0
Congeneric	2,2	19,0	22,5	2,6

The conspecific distance showed less than 2% divergence in 8 of the 14 species. Exceptionally deep sequence divergences were evident between individuals identified as the same morphospecies of *N*. *digrammus* (9.80% ± 1.0%), *N*. *trifasciatus* (8.1% ± 0.7%), *N*. *unifasciatus* (7.1% ± 0.7%), and *N*. *eques* (4.% ± 0.4%). We constructed a haplotype network based on Templeton’s method (95% statistical parsimony) [53], and all four species displayed unconnected networks, which was consistent with the clusters identified through the NJ tree. Each of these lineages or phylogroups is presented in more detail below.

### Nannostomus eques

**Fig 3:**
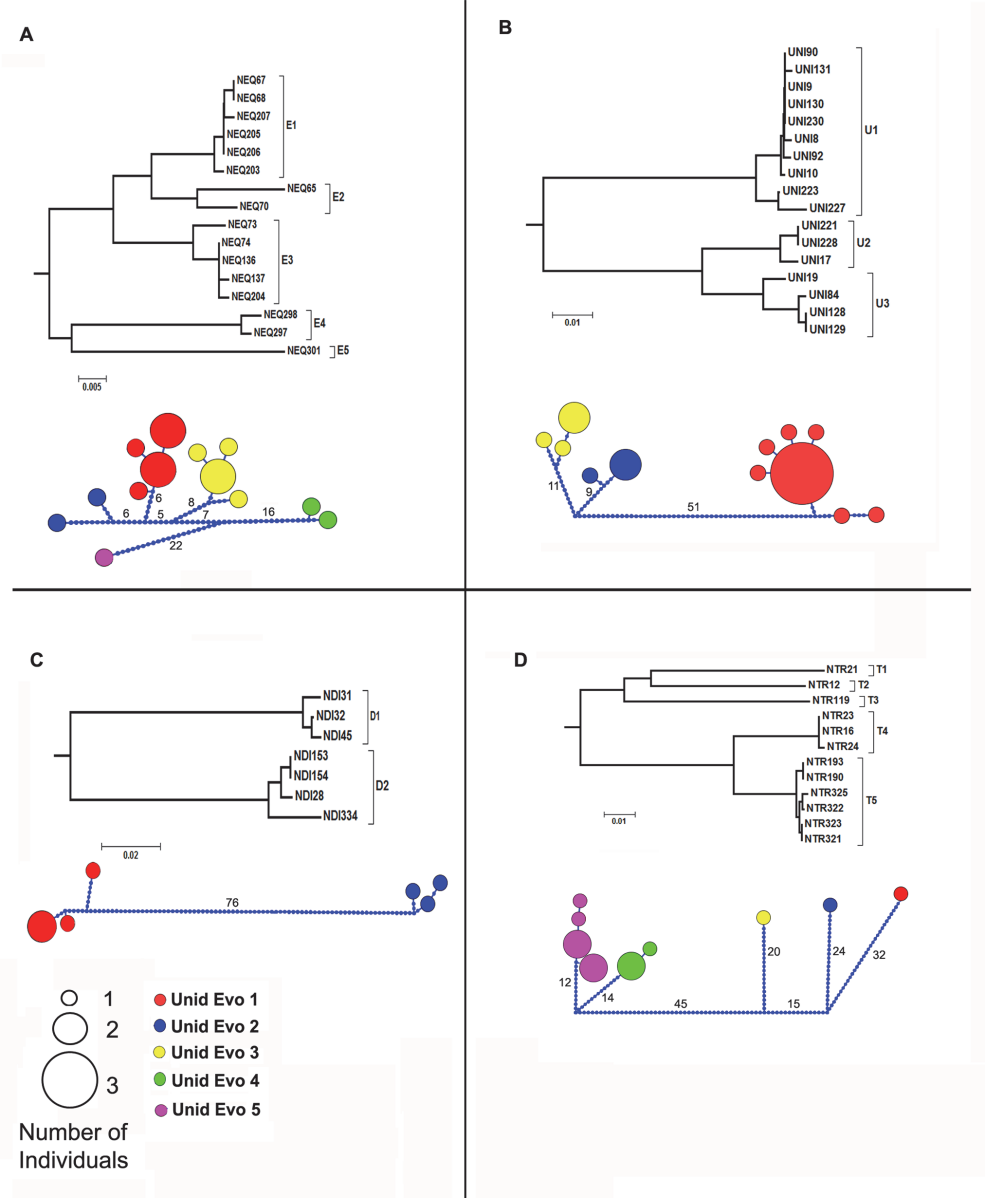
Neighbor-Joining tree and haplotype network of *Nannostomus* species showing distinct lineages: (A) *N*. *eques*, (B) *N*. *unifasciatus*, (C) *N*. *digrammus* and (D) *N*. *trifasciatus*. In the network each circle represents one haplotype, and theize of the circles is proportional to the haplotype frequency. Color codes represent the lineages found in *Nannostomus*. Numbers on the lines represent the mutational steps between haplotypes.

Of the 16 specimens identified as *N*. *eques*, 13 unique haplotypes were identified. These haplotypes were assigned to five lineages (E1—E5). E1 (n = 6) was sampled in Rio Tapajós, Lower Rio Amazonas and Lower Rio Negro, E2 (n = 2) in Upper Rio Amazonas and Upper Rio Negro: E3 (n = 5) in Lower Rio Amazonas and Upper Rio Negro, E4 (n = 3) in Rio Guamá, and E5 (n = 1) in Rio Madeira (Fig. 3A). Low genetic variation was observed within the lineages (E1 = 0.55%, E3 = 0.26%, E4 = 0.52%), with the exception of E2 (2.29%) (Table 2). The mean genetic distance between the lineages was 4.3%. The smallest genetic distance encompassing different lineages was found between E2 and E3 (3.4% ±0.7%), while the largest was between E1 and E5 (7. 7% ± 1.1%).

**Table 2:** Estimates of pairwise genetic distance between *Nannostomus* species under the Kimura 2- parameter (K2P) model.

	1	2	3	4	5	6	7	8	9	10	11	12	13	14	15	16	17	18	19	20	21	22	23
*1. N. beckfordi*	**0,911**	0,026	0,018	0,021	0,017	0,018	0,019	0,017	0,019	0,017	0,021	0,019	0,020	0,019	0,021	0,023	0,022	0,020	0,021	0,017	0,019	0,019	0,020
*2. N. britskii*	0,266	**0,103**	0,025	0,025	0,022	0,022	0,023	0,022	0,023	0,025	0,023	0,024	0,025	0,024	0,026	0,027	0,026	0,025	0,025	0,022	0,023	0,023	0,025
*3. N. digrammus1*	0,178	0,263	**1,501**	0,018	0,019	0,019	0,019	0,019	0,019	0,022	0,019	0,019	0,018	0,018	0,021	0,021	0,022	0,020	0,021	0,018	0,020	0,019	0,020
*4. N. digrammus2*	0,211	0,262	0,162	**0,813**	0,022	0,020	0,020	0,021	0,020	0,021	0,022	0,023	0,020	0,022	0,022	0,021	0,021	0,020	0,021	0,018	0,020	0,020	0,023
*5. N. eques1*	0,170	0,216	0,184	0,216	**0,557**	0,009	0,008	0,010	0,011	0,018	0,022	0,021	0,019	0,018	0,021	0,022	0,021	0,022	0,022	0,018	0,019	0,019	0,020
*6. N. eques2*	0,177	0,227	0,188	0,196	0,048	**2,293**	0,007	0,011	0,011	0,017	0,021	0,021	0,018	0,016	0,020	0,022	0,021	0,021	0,021	0,017	0,019	0,019	0,019
*7. N. eques3*	0,186	0,226	0,191	0,191	0,041	0,034	**0,265**	0,011	0,011	0,018	0,022	0,021	0,019	0,017	0,021	0,022	0,021	0,022	0,023	0,018	0,019	0,018	0,020
*8. N. eques4*	0,159	0,214	0,186	0,207	0,066	0,076	0,072	**0,521**	0,011	0,018	0,021	0,021	0,019	0,018	0,022	0,023	0,021	0,020	0,021	0,018	0,018	0,018	0,018
*9. N. eques5*	0,171	0,232	0,174	0,182	0,077	0,076	0,074	0,072	**0,000**	0,019	0,021	0,021	0,019	0,018	0,020	0,022	0,021	0,021	0,023	0,017	0,020	0,019	0,020
*10. N. harrisoni*	0,153	0,266	0,225	0,217	0,177	0,179	0,182	0,182	0,190	**0,000**	0,022	0,022	0,021	0,020	0,021	0,021	0,020	0,021	0,021	0,017	0,021	0,021	0,020
*11. N. marginatus*	0,214	0,241	0,189	0,215	0,217	0,214	0,214	0,206	0,202	0,224	**1,252**	0,016	0,021	0,022	0,022	0,023	0,024	0,020	0,020	0,022	0,022	0,022	0,015
*12. N. mortenthaleri*	0,186	0,258	0,188	0,228	0,196	0,201	0,201	0,197	0,199	0,218	0,126	**0,000**	0,022	0,021	0,025	0,023	0,024	0,023	0,023	0,023	0,022	0,021	0,016
*13. N. marylinae*	0,188	0,260	0,170	0,178	0,182	0,174	0,182	0,188	0,178	0,207	0,224	0,215	**0,642**	0,019	0,021	0,021	0,022	0,021	0,023	0,021	0,022	0,020	0,022
*14. N. nitidus*	0,183	0,245	0,178	0,219	0,173	0,147	0,158	0,172	0,184	0,195	0,229	0,208	0,190	**0,881**	0,024	0,024	0,023	0,022	0,023	0,020	0,021	0,020	0,022
*15. N. trifasciatus1*	0,221	0,283	0,210	0,216	0,221	0,218	0,218	0,229	0,214	0,211	0,239	0,269	0,216	0,245	**0,000**	0,015	0,016	0,017	0,018	0,022	0,023	0,023	0,024
*16. N. trifasciatus2*	0,240	0,293*	0,215	0,216	0,226	0,229	0,232	0,233	0,228	0,213	0,248	0,239	0,205	0,253	0,106	0,000	0,014	0,019	0,017	0,022	0,022	0,021	0,025
*17. N. trifasciatus3*	0,227	0,283	0,210	0,199	0,202	0,208	0,208	0,207	0,214	0,207	0,257	0,253	0,223	0,229	0,128	0,115	0,000	0,018	0,018	0,022	0,022	0,023	0,024
*18. N. trifasciatus4*	0,195	0,289	0,199	0,208	0,219	0,214	0,231	0,199	0,214	0,211	0,209	0,242	0,216	0,240	0,152	0,162	0,149	0,115	0,010	0,023	0,024	0,023	0,023
*19. N. trifasciatus5*	0,209	0,286	0,204	0,212	0,231	0,228	0,240	0,213	0,238	0,211	0,217	0,240	0,234	0,238	0,153	0,140	0,148	0,050	0,335	0,021	0,023	0,022	0,022
*20. N. unifasciatus1*	0,162	0,237	0,192	0,183	0,178	0,168	0,173	0,160	0,166	0,162	0,226	0,226	0,210	0,187	0,224	0,224	0,230	0,233	0,226	0,715	0,015	0,015	0,021
*21. N. unifasciatus2*	0,188	0,245	0,213	0,205	0,187	0,184	0,188	0,185	0,195	0,211	0,237	0,221	0,212	0,199	0,235	0,229	0,234	0,252	0,245	0,124	0,579	0,008	0,023
*22. N. unifasciatus3*	0,183	0,239	0,199	0,205	0,184	0,180	0,176	0,178	0,189	0,203	0,222	0,211	0,203	0,192	0,233	0,226	0,247	0,244	0,236	0,125	0,046	0,931	0,021
*23. N. rubrotaniatus*	0,199	0,255	0,214	0,230	0,196	0,196	0,191	0,165	0,190	0,192	0,130	0,123**	0,225	0,225	0,249	0,265	0,245	0,239	0,235	0,206	0,232	0,214	0,000

Pairwise congeneric divergence is denoted by the number of base substitutions per site between species (below diagonal) with their corresponding standard error (above diagonal). Complete deletion of all codon positions (1^st^, 2^nd^, 3^rd^ and noncoding) was employed in this analysis. Mean conspecific divergences (%) are listed on the diagonal in bold italics.

*Large congeneric K2P distance;

**Small congeneric K2P distance.

### Nannostomus unifasciatus

Of the 17 specimens identified as *N*. *unifasciatus*, 13 unique haplotypes were identified. These haplotypes were assigned to three lineages (U1—U3); U1 (n = 10) was sampled in Rio Purus, Rio Amazonas, and Lower and Middle Rio Negro. U2 (n = 3) in Lower and Middle Rio Negro and U3 (n = 4) in Middle and Upper Rio Negro (Fig. 3B). Moderate genetic variation was observed within the U1, U2 and U3 lineages (0.71%, 0.57% and 0.93%, respectively). The mean genetic distance between the lineages was 7.1%. The smallest genetic distance comprising different lineages was found between U2 and U3 (4.6% ± 0.8%), while the greatest was between U1 and U3 (12.5% ± 1.4%) (Table 2).

### Nannostomus digrammus

Of the 7 specimens identified as *N*. *digrammus*, 6 unique haplotypes were identified. These haplotypes were assigned to lineages (D1 and D2). D1 (n = 3) was sampled in the Tapajós River, and Amazonas and Takutu River tributaries, while D2 (n = 3) was endemic to the Negro River (Fig. 3C). Moderate genetic variation was seen within the D1 and D2 lineages (1.50% and 0.80%, respectively) (Table 2). The mean genetic distance between the lineages was 16.2% ± 1.9.

### Nannostomus trifasciatus

Of the 12 specimens identified as *N*. *trifasciatus*, 9 unique haplotypes were identified. These haplotypes were assigned to 5 lineages (T1—T5). T1 (n = 1) was sampled in Rio Preto da Eva, T2 (n = 1) in Upper Rio Negro, T3 (n = 1) in Upper Rio Negro, T4 (n = 3) in Middle Rio Negro, and T5 (n = 6) in tributaries of Rio Takutu, Uatumã, and Amazonas near Macapá City (Fig. 3D). Low genetic variation was observed within T4 and T5 (0.11% and 0.33%, respectively) (Table 2). The mean genetic distance between the lineages was 8.1%. The smallest genetic distance encompassing different lineages was found between T4 and T5 (5.0% ± 1.0%), while the largest was between T2 and T4 (16.2% ± 1.9%).

## Discussion

Molecular methodologies developed rapidly in recent years, and currently DNA barcoding is considered the most useful tool for species identification. Indeed, a great advantage offered by DNA barcoding is the possibility of identifying cryptic species, as can be seen in several publications since its launch in 2003 [54].

According to the Fish Barcode of Life project database (www.fishbol.org), only one *Nannostomus* species had been previously barcoded. In our data set, 14 *Nannostomus* species were DNA barcoded, and they were easily identified by this approach, given that all recognized species formed monophyletic clusters (Fig. 2). However, two species (*N*. *nitidus* and *N*. *limatus*) revealed shallow interspecific sequence divergence (2.2%) when compared to other *Nannostomus* species (Table 1). Despite this, no evidence of shared sequences among both species were observed, suggesting either recent speciation or the need of synonymization.

In contrary, four species (*N*. *digrammus*, *N*. *trifasciatus*, *N*. *unifasciatus*, and *N*. *eques*) showed deep intraspecific sequence divergence, suggesting the existence of overlooked species within *Nannostomus*, although some lineages were represented by a single individual (Table 2).

The *COI* delineations of the T1, T2, T3 and E5 *Nannostomus* lineages were achieved with only one individual. We are aware that hypothetical intraspecific lineages can be hindered by inadequate sample size in large geographic areas such as the Amazon Basin. In flathead fishes, for example, the limited number of specimens in a particular lineage and the sparse geographical spread of the samples for some of the proposed lineages restricted the ability to evaluate the extent of genetic diversity across several groups [55]. Thus, we suggest future surveys of these *Nannostomus* lineages to confirm if they do, in fact, represent evolutionary units.

All the lineages found in each of the above mentioned species were well supported by bootstrap values (>80) in the NJ tree, independent of the mutational steps necessary to connect the haplotype networks below the standard statistical parsimony (Figs. 2 and 3). The two lineages observed in *N*. *digrammus* (D1 and D2) diverged by 12.6%. The five lineages of *N*. *trifasciatus* had a mean divergence of 8.1%, while that of the three lineages of *N*. *unifasciatus* was of 7.1%. Finally, the five lineages of *N*. *eques* diverged by 4.3% (mean). The divergence between the lineages of *Nannostomus* was greater than obtained for other marine and freshwater fish species [20, 56–61].

It was evident in this study that the cutoff value of 2% does not apply to *Nannostomus* species, given the mean congeneric (∼19%) and conspecific (∼3%) distances were high. Indeed, surveys on North American and Neotropical freshwater ichthyofauna have shown that the mean congeneric and conspecific genetic distances are usually > 6.8% and <0.73%, respectively [62–63], with the exception of the Pampa Plain freshwater fishes at, the southernmost distribution range of many Neotropical species, where the mean congeneric genetic distance is 1.67% [64].

Among lebiasinids, only *Nannostomus* has twice been taxonomically revised, in addition to the phylogenetic hypotheses [32–34,36]. Morphology-based taxonomy has shown that *Nannostomus* cannot be considered to have a phenotypic conservatism. Apparently, there are poorly defined *Nannostomus* species complexes based on morphological grounds, including *N*. *beckfordi*, *N*. *eques*, *N marginatus*, and *N*. *trifasciatus* (A. L. Netto-Ferreira, unpublished data). Recent mitochondrial and nuclear DNA data have revealed hypothetical evolutionarily significant units in species of *Nannostomus*. For example, in *N*. *unifasciatus*, the DNA sequence data of the intron in the S7 ribosomal protein gene revealed two distinct lineages in the Rio Negro basin [43]. In *N*. *eques*, the mitochondrial DNA control region also revealed the existence of two lineages in the Rio Negro basin [44]. Together, the morphological and genetic studies indicate that species richness in the genus is probably underestimated.

The Amazon aquatic ecosystem is rich in diversity and quick access to information on Amazonian biodiversity is essential. Forest degradation and, fishery over-exploitation enhance the risks of species extinction, and quickly updated information regarding the fishes caught in wild fisheries (such as the *Nannostomus* species) is necessary to implement appropriate practices for to their conservation and, management and to prevent exploitation.

Pencilfishes are commercial ornamental fish and constitute a source of revenue for the riverine people of the Amazon. The discovery of cryptic species becomes very important when they are targets for commercial use. Considering the economic importance of the *Nannostomus* species, its DNA barcoding contributes to conservation policy in two important ways: by enhancing Amazonian biodiversity assessments to prioritize conservation areas (e.g., upper Rio Negro), and by providing information about evolutionary histories and phylogenetic diversity (e.g., unveiling hidden diversity).

DNA barcoding of ornamental marine fishes has generated data and provided confidence in species identification, opening new avenues for managing business practices [65]. Of the approximately ∼500 Brazilian ornamental fishes allowed to be sold in the ornamental fish trade, 7 are pencilfishes: *N*. *beckfordi*, *N*. *digrammus*, *N*. *eques*, *N*. *espei*, *N*. *marginatus*, *N*. *trifasciatus*, and *N*. *unifasciatus*. Our study contributes new Amazonian fish barcodes, providing for a more comprehensive species identification of the ornamental pencilfishes, in addition to revealing the hidden diversity in the analyzed *Nannostomus* species. It is certain that future species delimitation of *Nannostomus* should be in accordance with the spirit of integrative taxonomy as well and based on congruence across analyses that utilize multiple data sources.
