# Correction: DNA Barcoding in Pencilfishes (Lebiasinidae: *Nannostomus*) Reveals Cryptic Diversity across the Brazilian Amazon

**DOI:** 10.1371/journal.pone.0123363

**Published:** 2015-04-02

**Authors:** The PLOS ONE Staff

Table 2 is missing rows for *N*. *espei*, *N*. *limatus*, and line 17 incorrectly contains the name *N*. *rubrotaniatus* instead of *N*. *rubrocaudatus*. The authors have provided a correct version of Table 2 here.

**Table 2:** Estimates of pairwise genetic distance between *Nannostomus* species under the Kimura 2- parameter (K2P) model.

	1	2	3	4	5	6	7	8	9	10	11	12	13	14	15	16	17	18	19	20	21	22	23	24	25
*1*. *N*. *beckfordi*	***0*,*911***	0,026	0,018	0,021	0,018	0,019	0,019	0,017	0,019	0,019	0,017	0,020	0,021	0,019	0,019	0,019	0,020	0,021	0,022	0,021	0,020	0,020	0,018	0,019	0,020
*2*. *N*. *britskii*	0,266	***0*,*103***	0,024	0,024	0,022	0,022	0,023	0,022	0,023	0,022	0,026	0,025	0,022	0,024	0,024	0,024	0,024	0,026	0,026	0,026	0,026	0,026	0,022	0,022	0,023
*3*. *N*. *digrammus1*	0,178	0,263	***1*,*501***	0,018	0,019	0,019	0,019	0,018	0,018	0,018	0,021	0,018	0,019	0,019	0,017	0,018	0,020	0,020	0,021	0,021	0,020	0,021	0,018	0,019	0,020
*4*. *N*. *digrammus2*	0,211	0,262	0,162	***0*,*813***	0,021	0,020	0,019	0,020	0,020	0,018	0,021	0,022	0,021	0,023	0,019	0,022	0,022	0,021	0,021	0,021	0,021	0,021	0,018	0,020	0,020
*5*. *N*. *eques1*	0,170	0,216	0,184	0,216	***0*,*557***	0,008	0,008	0,010	0,011	0,017	0,018	0,019	0,021	0,020	0,018	0,018	0,020	0,021	0,023	0,020	0,022	0,022	0,018	0,018	0,019
*6*. *N*. *eques2*	0,177	0,227	0,188	0,196	0,048	***2*,*293***	0,007	0,011	0,011	0,018	0,018	0,016	0,020	0,020	0,018	0,016	0,019	0,020	0,022	0,020	0,021	0,021	0,017	0,018	0,019
*7*. *N*. *eques3*	0,186	0,226	0,191	0,191	0,041	0,034	***0*,*265***	0,010	0,011	0,018	0,018	0,017	0,021	0,020	0,018	0,017	0,019	0,021	0,022	0,021	0,023	0,023	0,018	0,017	0,019
*8*. *N*. *eques4*	0,159	0,214	0,186	0,207	0,066	0,076	0,072	***0*,*521***	0,010	0,017	0,018	0,018	0,020	0,021	0,019	0,018	0,018	0,022	0,023	0,020	0,021	0,022	0,018	0,018	0,018
*9*. *N*. *eques5*	0,171	0,232	0,174	0,182	0,077	0,076	0,074	0,072	***0*,*000***	0,019	0,019	0,019	0,020	0,021	0,018	0,018	0,020	0,020	0,022	0,021	0,021	0,023	0,017	0,019	0,020
*10*. *N*. *espei*	0,173	0,224	0,177	0,172	0,170	0,176	0,180	0,167	0,191	***0*,*000***	0,018	0,017	0,020	0,019	0,018	0,017	0,021	0,019	0,019	0,019	0,021	0,021	0,018	0,019	0,020
*11*. *N*. *harrisoni*	0,153	0,266	0,225	0,217	0,177	0,179	0,182	0,182	0,190	0,172	***0*,*000***	0,020	0,021	0,021	0,021	0,020	0,019	0,021	0,021	0,020	0,021	0,021	0,017	0,021	0,021
*12*. *N*. *limatus*	0,188	0,253	0,179	0,228	0,183	0,155	0,168	0,172	0,192	0,145	0,193	***0*,*115***	0,022	0,021	0,019	0,006	0,022	0,024	0,023	0,022	0,023	0,023	0,019	0,020	0,021
*13*. *N*. *marginatus*	0,214	0,241	0,189	0,215	0,217	0,214	0,214	0,206	0,202	0,223	0,224	0,237	***1*,*252***	0,016	0,021	0,021	0,015	0,022	0,022	0,023	0,019	0,020	0,021	0,021	0,022
*14*. *N*. *mortenthaleri*	0,186	0,258	0,188	0,228	0,196	0,201	0,201	0,197	0,199	0,181	0,218	0,207	0,126	***0*,*000***	0,021	0,021	0,015	0,024	0,022	0,023	0,023	0,022	0,022	0,021	0,021
*15*. *N*. *marylinae*	0,188	0,260	0,170	0,178	0,182	0,174	0,182	0,188	0,178	0,176	0,207	0,197	0,224	0,215	***0*,*642***	0,019	0,021	0,021	0,021	0,022	0,021	0,022	0,020	0,020	0,021
*16*. *N*. *nitidus*	0,183	0,245	0,178	0,219	0,173	0,147	0,158	0,172	0,184	0,145	0,195	***0*,*022*** *	0,229	0,208	0,190	***0*,*881***	0,021	0,023	0,024	0,022	0,023	0,023	0,019	0,020	0,020
*17*. *N*. *rubrocaudatus*	0,199	0,255	0,214	0,230	0,196	0,196	0,191	0,165	0,190	0,207	0,192	0,229	0,130	0,123	0,225	0,225	***0*,*000***	0,023	0,025	0,023	0,023	0,022	0,021	0,021	0,022
*18*. *N*. *trifasciatus1*	0,221	0,283	0,210	0,216	0,221	0,218	0,218	0,229	0,214	0,192	0,211	0,255	0,239	0,269	0,216	0,245	0,249	***0*,*000***	0,014	0,016	0,017	0,017	0,021	0,022	0,022
*19*. *N*. *trifasciatus2*	0,240	***0*,*293*** *	0,215	0,216	0,226	0,229	0,232	0,233	0,228	0,189	0,213	0,247	0,248	0,239	0,205	0,253	0,265	0,106	***0*,*000***	0,014	0,018	0,017	0,021	0,021	0,022
*20*. *N*. *trifasciatus3*	0,227	0,283	0,210	0,199	0,202	0,208	0,208	0,207	0,214	0,190	0,207	0,225	0,257	0,253	0,223	0,229	0,245	0,128	0,115	***0*,*000***	0,018	0,018	0,022	0,023	0,022
*21*. *N*. *trifasciatus4*	0,195	0,289	0,199	0,208	0,219	0,214	0,231	0,199	0,214	0,210	0,211	0,238	0,209	0,242	0,216	0,240	0,239	0,152	0,162	0,149	***0*,*115***	0,009	0,023	0,023	0,024
*22*. *N*. *trifasciatus5*	0,209	0,286	0,204	0,212	0,231	0,228	0,240	0,213	0,238	0,213	0,211	0,234	0,217	0,240	0,234	0,238	0,235	0,153	0,140	0,148	0,050	***0*,*335***	0,022	0,022	0,023
*23*. *N*. *unifasciatus1*	0,162	0,237	0,192	0,183	0,178	0,168	0,173	0,160	0,166	0,174	0,162	0,188	0,226	0,226	0,210	0,187	0,206	0,224	0,224	0,230	0,233	0,226	***0*,*715***	0,015	0,015
*24*. *N*. *unifasciatus2*	0,183	0,239	0,199	0,205	0,184	0,180	0,176	0,178	0,189	0,190	0,203	0,194	0,222	0,211	0,203	0,192	0,214	0,233	0,226	0,247	0,244	0,236	0,125	***0*,*579***	0,008
*25*. *N*. *unifasciatus3*	0,188	0,245	0,213	0,205	0,187	0,184	0,188	0,185	0,195	0,194	0,211	0,206	0,237	0,221	0,212	0,199	0,232	0,235	0,229	0,234	0,252	0,245	0,124	0,046	***0*,*931***

Pairwise congeneric divergence is denoted by the number of base substitutions per site between species (below diagonal) with their corresponding standard error (above diagonal). Complete deletion of all codon positions (1^st^, 2^nd^, 3^rd^ and noncoding) was employed in this analysis.

Mean conspecific divergences (%) are listed on the diagonal in bold italics.

*Large congeneric K2P distance;

**Small congeneric K2P distance.
